# Infective Endocarditis in a Young Adult due to *Lactococcus lactis*: A Case Report and Review of the Literature

**DOI:** 10.1155/2018/5091456

**Published:** 2018-03-04

**Authors:** G. Georgountzos, C. Michopoulos, C. Grivokostopoulos, M. Kolosaka, N. Vlassopoulou, A. Lekkou

**Affiliations:** ^1^Faculty of Medicine, Department of Health Sciences, University of Patras, Rio, Patras 26504, Greece; ^2^Department of Internal Medicine, Patras University Hospital, Rio, Patras 26504, Greece; ^3^Department of Cardiology, Patras University Hospital, Rio, Patras 26504, Greece; ^4^Department of Infectious Diseases, Patras University Hospital, Rio, Patras 26504, Greece

## Abstract

Infective endocarditis (IE) is a condition mainly associated with valvular disease or prosthetic valve and intravenous drug use as a risk factor. Here, we describe a rare case of a previously healthy patient with endocarditis due to *Lactococcus lactis* associated with cattle contact, where antibiotic treatment resulted in full recovery.

## 1. Introduction

The *Lactococcus* genus has two main representatives, *L. lactis* and *L. cremoris*. *Lactococcus lactis* is an anaerobic, catalase-negative, and Gram-positive microorganism widely used in cheese production and dairy milk products like yogurt and sour cream. *L. lactis* is considered to have low virulence and pathogenic potential although it has been associated with some diseases in healthy, immunocompetent, or immunocompromised patients. A few cases including infective endocarditis (IE) in adults and in children have been documented. Herein, we describe a case of infective endocarditis in a young adult due to *Lactococcus lactis*.

## 2. Case Presentation

A 35-year-old blacksmith presented at the emergency department with a 3-week history of fever to 38.8°C, chills, weakness, and night sweats. The patient denied the existence of shortness of breath, chest pain, vomiting, diarrhea, abdominal pain, dysuria, and any other symptoms. His previous medical history was clear, and he mentioned no use of cigarettes, alcohol, unprocessed dairy products, or intravenous drugs. In the past, he had dental implants, and his last visit at the dentist was 6 months ago. He lives in a village, where he takes care of some chickens, rabbits, and lambs. Also, one week prior to his hospital admission, he visited his family doctor who suggested the administration of levofloxacin (500 mg/d per os) for ten days.

On admission, he was in a good level of consciousness with blood pressure of 150/76 mmHg, atmospheric air oxygen saturation of 99%, and heart rate at 93 beats/min. On clinical examination, a diastolic murmur was audible at the second right intercostal space (aortic valve area). No pathologic findings were found on lung and abdominal examination. No other mucocutaneous signs of endocarditis were observed.

**Figure 1:**
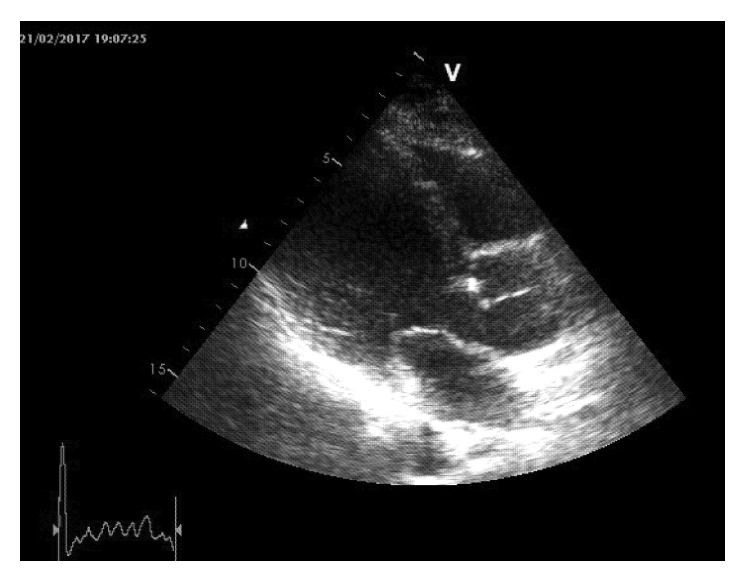
Transthoracic ultrasound showing a degenerated bicuspid aortic valve, combined with left ventricle dilation. A small mass on the left aortic cusp is distinguished as septic vegetation, due to *L. lactis*.

**Figure 2:**
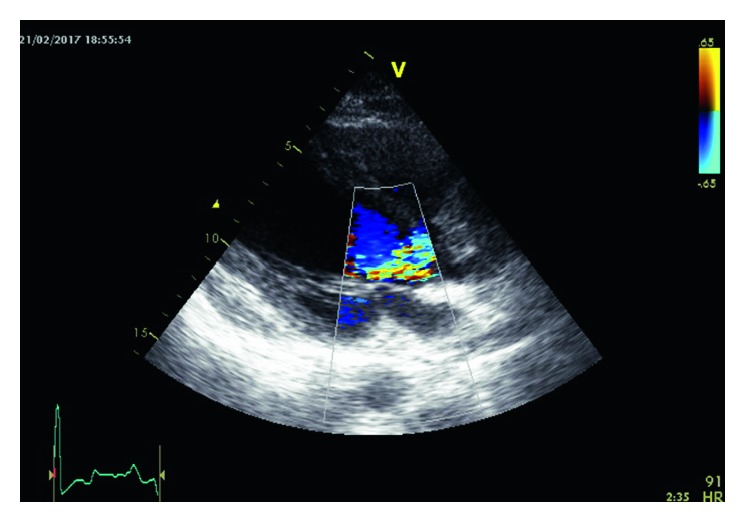
Doppler echocardiography and colour flow mapping revealed moderate-to-severe (3/4) aortic valve failure.

Blood tests showed CRP of 2.5 mg/dl, erythrocyte sedimentation rate (ESR) of 20 mm/h, and white blood cell (WBC) count of 12.000/*μ*L. Urine analysis and urine culture were negative. The chest X-ray, the electrocardiography, and the ultrasound of the abdomen were normal. Two sets (aerobic and anaerobic) of blood cultures were obtained with a difference of 20 minutes between them, and a total of 4 sets of blood cultures were finally received. The patient was initially treated with intravenous levofloxacin 500 mg and ceftriaxone 2 g per day. The serum immunological investigation for HIV, HAV, HBV, HCV, CMV, *Toxoplasma*, EBV, HSV, Coxsackie, RF, C_3_, C_4_, ANA, ANCA, *Coxiella burnetii*, *Leishmania*, *Leptospira*, and *Brucella* was found to be negative. The ophthalmologic examination with fundoscopy had no findings. Transthoracic and transesophageal echocardiography showed left ventricle dilation (ejection fraction 65%) and a degenerated bicuspid aortic valve with a small vegetation (0.5 × 0.6 cm) at the left cusp and a moderate-to-severe regurgitation. These findings are well depicted in Figures 1 and 2. Two blood cultures defined the underlying pathogen, which was *Lactococcus lactis*, all in anaerobic bottles. The isolate was sensitive to ampicillin, ceftriaxone, clindamycin, chloramphenicol, erythromycin, oxacillin, teicoplanin, and vancomycin. An alternative antimicrobial therapy with ceftriaxone 2 g q.d. and gentamicin 80 mg t.i.d. was initiated. During the following days, the inflammation markers dropped (CRP: 0.55 and WBC 7.53 × 10^3^/*μ*L), and from the fourth day of antibiotic treatment, the patient was afebrile. Two sets of blood cultures were obtained whose results were negative. On hospital day 10, administration of gentamicin was ended, and a 6-week course of intravenous ceftriaxone was given. A new transthoracic echocardiography revealed a further decrease in vegetation size. In 3 months of follow-up, the patient was symptom-free.

## 3. Discussion

Taking into consideration the clinical presentation and the further investigation (persistent fever, endocardial involvement documented by transthoracic and transesophageal ultrasound, bicuspid aortic valve, and sustained bacteremia), the patient was diagnosed with IE, according to the modified Duke criteria [1]. There are only few cases with lactococcal IE; thus, the therapeutic protocol was based on the susceptibility of the lactococcal isolate.

*Lactococcus lactis* (former *Streptococcus lactis*) is a spherical-shaped mesophilic, microaerophilic fermenting bacterium. Among the subspecies of *Lactococcus* species, the main bacteria are *L. lactis* and *L. cremoris*, which seem to be skin commensals in the cattle. They are really popular in dairy industry because of their use for cheese and fermented milk products. Lately, their reputation is also growing in the vaccine industry, and there is research in animal models supporting vaccination for infectious diseases such as avian flu and pneumococcal infections [2, 3]. *Lactococcus lactis* has low virulence, and in general, it is nonpathogenic, but recently, it is considered as the opportunistic pathogen microbe. A possible condition affecting the virulence and the infectious potential of *Lactococcus lactis* in infective endocarditis is its ability of heterologous expression of surface glycoprotein Cnm which promotes adherence to cardiac tissue [4]. There are some reports that indicate the involvement of *L. lactis* in emergency situations such as septic arthritis [5], liver and cerebral abscess [6, 7], peritonitis [8], and osteomyelitis [9]. These reports include immunocompetent and immunocompromised patients.

Lactococcal IE is very rare, and only few cases in adults and fewer cases in children/infants can be found in the literature [10–19]. These cases are summarized in Table 1. In 2 cases [15], including ours, the affected valve was the aortic valve, which was bicuspid. However, in other cases, endocarditis seemed to attack the atrioventricular valves [10–14, 16–19].

Rheumatic valvular disease or other valvular impairment, prosthetic valve, intravenous drug use, and congenital heart disease are well-documented lifetime-predisposing factors for IE. However, only five patients with lactococcal IE including ours [11, 14, 15, 17] had a predisposing condition associated with high risk of IE. In the rest of them, there was not a history of valvular heart disease [10, 12, 13, 16, 18, 19].

*L. lactis* is not considered a human pathogen, and human infections in people with immunosuppresion or impaired defence mechanisms are opportunistic infections. Due to the rarity of the *Lactococcus* infection, the route of this infection is not well demonstrated. The hypotheses about the source of infection include exposure by ingestion or contact with unpasteurized dairy products or raw milk [10, 13, 15]. Another proposed mechanism which is also suggested for our patient, who was occupied with sheep, chickens, and rabbits, is direct intraluminal spreading from contaminated hands. However, many patients declared that they did not consume any dairy products or raw milk [7, 11, 12, 14, 16, 17, 19].

From the literature, we can easily assume that this condition might be associated with severe complications. Most of the patients developed cerebral embolism, and in one of them, this caused hemorrhage [16] and death. Another patient developed multiple mycotic aneurysms [15], and there is also a case of young child presenting with pulmonary embolization [19]. Another reported complication was cardiac arrhythmia due to excessive inflammation of the conduction system of the heart in an infant who did not survive the infection [18].Our patient recovered and was free of complications in 3 months of follow-up.

## 4. Conclusion

Lactococcal endocarditis is an extremely rare condition that can be presented at any age. Despite its low virulence and pathogenic potential, *Lactococcus* should be treated as serious infection because of the 2 referred deaths and its complications. It is essential for the clinician to suspect lactococcal endocarditis, when he notices (1) long-lasting fever with (2) newly audible murmur or (3) cerebrovascular event in a patient with a (4) history of unpasteurized dairy product consumption. As long as there is no specific guidance, antimicrobial therapy should be in compliance with the susceptibility of the pathogen isolated from the cultures. Further cases need to be reported and analyzed to develop a therapeutic and preventive strategy for this disease.

## Figures and Tables

**Table 1:** Reported cases of *Lactococcus lactis*–associated infective endocarditis.

References	Heart disease	Complications	Clinical outcome
Wood et al. [10]	No history of heart disease	—	Recovered
Mannion and Rothburn [11]	Rheumatic valve disease	Infarction/dysphasia	Recovered
Pellizzer et al. [12]	No history of heart disease	—	Recovered
Halldorsdottir et al. [13]	No history of heart disease	—	Recovered
Zechini et al. [14]	Atrial myxoma and mitral regurgitation	—	Recovered after surgery
Resch et al. [15]	Bicuspid aortic valve	Multiple mycotic aneurysms	Recovered after surgery
Lin et al. [16]	No history of heart disease	Intracerebral hemorrhage/infraction	Deceased
Rostagno et al. [17]	Bileaflet mitral valve prolapse (prosthetic valve repair)	Embolic infraction	Recovered after surgery
Taniguchi et al. [18]	No history of heart disease	Arrhythmia (severe inflammation of the conductive system)	Deceased
Mansour et al. [19]	No history of heart disease	Pulmonary septic emboli	Recovered
